# Assessing Bias in Medication Administration in Relation to Incarceration History in a New Orleans Student Clinic Population

**DOI:** 10.1007/s10900-026-01633-3

**Published:** 2026-09-03

**Authors:** Haley Watts, Diane Hurwitz, Jonathan Hurley, Ellen Terry, Christopher Saker, Thomas Stall, Vidhatri Raturi, Anjali Niyogi, Helen Pope

**Affiliations:** 1 Deming Department of Medicine, Tulane University School of Medicine, New Orleans, LA USA; 2 Formerly Incarcerated Transitions (FIT) Clinic, New Orleans, LA USA; 3 Department of Medicine, Johns Hopkins University School of Medicine, Baltimore, MD USA; 4 Department of Orthopaedic Surgery, Walter Reed National Military Medical Center, Bethesda, MD USA

**Keywords:** Carceral health, Healthcare bias, Formerly incarcerated, Student-run clinics

## Abstract

Formerly incarcerated individuals experience a disproportionate burden of disease and face greater barriers to accessing healthcare. This population also represents a significant proportion of patients seen in New Orleans’ student-run clinics. This study examines the chief complaints among formerly incarcerated patients and evaluates whether prescribing patterns differ for this group, with the aim of identifying potential biases in treatment practices. Data for this study were obtained from REDCap surveys administered to patients at student-run clinics. Patients were categorized by chief complaint, and chi-square analysis was used to assess whether incarceration status was associated with initiation of the corresponding treatment. Analyses were performed in SPSS, with p-values < 0.05 considered statistically significant. Logistic regression was subsequently conducted to evaluate potential confounders and to confirm associations identified in the chi-square analysis. No significant differences were observed in overall chief complaints or overall medication prescribing between formerly incarcerated and non-incarcerated patients. However, after adjusting for demographic factors, formerly incarcerated patients presenting with respiratory symptoms were less likely to receive cough and cold medications. These findings suggest a potential bias in prescribing practices, as formerly incarcerated patients presenting with respiratory symptoms were less likely to receive cough and cold medications compared to their non-incarcerated counterparts.

## Introduction

In 2021, Louisiana had the highest incarceration rate in the United States, with 963 individuals imprisoned per 100,000 people, nearly three times the national average of 359 per 100,000 [1, 2]. Within Louisiana, Orleans Parish, home to the city of New Orleans, there is an imprisonment rate of 652 per 100,000. Orleans Parish also has the highest number of residents in state custody, with 2,519 individuals incarcerated under the Louisiana Department of Public Safety & Corrections in 2022. Notably, 19 of the city’s 72 neighborhoods have imprisonment rates of over 1,000 per 100,000, indicating that at least 1% of residents in these communities are incarcerated [3]. Given the high rates of incarceration and the large proportion of formerly incarcerated individuals in the New Orleans community, healthcare providers in New Orleans frequently care for individuals with a history of incarceration and must understand the unique health and social needs of this patient population.

Incarceration is widely recognized to adversely affect both individual and community health. A 2019 analysis of U.S. counties demonstrated that higher incarceration rates are associated with increased morbidity (self-reported fair or poor health) and mortality [4]. Incarcerated individuals have a higher prevalence of chronic conditions including hypertension, asthma, arthritis, and infectious diseases such as hepatitis C, HIV, and tuberculosis when compared to the general population [5, 6, 7, 8]. Additionally, nearly half (44%) of incarcerated individuals report a history of mental health disorders, with many experiencing significant psychological distress during incarceration [9]. Despite this elevated disease burden, evidence suggests that these chronic conditions are often undertreated. One study found that the distribution of prescription medications for chronic illnesses such as diabetes, asthma, hypertension, hepatitis B or C, HIV, depression, and severe mental illness was significantly lower among incarcerated individuals compared to non-incarcerated individuals [10].

Following release, these individuals continue to experience significant barriers to accessing healthcare. These barriers include the loss of health insurance coverage while incarcerated and inadequate discharge planning, such as being released without discharge medications or follow-up appointments [11]. Compared to those without an incarceration history, formerly incarcerated individuals are less likely to have a primary care physician and less likely to receive preventive care services, including blood pressure monitoring, cholesterol screening, glucose testing, and breast cancer and colon cancer screening [12].

Social and structural challenges further compound these disparities. Formerly incarcerated individuals are nearly ten times more likely to experience homelessness than the general population [13]. In New Orleans, homelessness has been steadily rising, with 1,454 citizens experiencing homelessness as of January 2024, 66% of whom reside in shelters [14]. Communities with high rates of incarceration are also disproportionately affected by socioeconomic disadvantage and are more likely to include Black and Hispanic populations [15].

Student-run free clinics in New Orleans play an important role in addressing the gaps in care detailed above. These clinics operate primarily within homeless shelters and community centers where they provide a variety of services including primary care, blood pressure and blood glucose screenings, and testing for infectious diseases such as tuberculosis, HIV, and hepatitis C.

Due to the significant disease burden and persistent barriers to accessing care reported throughout the literature, this non-comparative study aims to evaluate the chief complaints of formerly incarcerated patients who present to student-run clinics. A secondary objective is to evaluate medication prescribing patterns in relation to these complaints. By examining potential differences in treatment, this study seeks to address a gap in the literature regarding post-incarceration prescribing practices and to identify potential biases in care. The investigators hope that these findings may inform more equitable and effective healthcare delivery for this vulnerable patient population.

## Methods

In collaboration with the Student Clinic Council (SCC), data were obtained from surveys administered across more than 30 student-run clinic sites affiliated with a medical school in New Orleans. The survey, developed by the SCC executive committee, functions as a standardized data collection tool in the absence of an electronic medical record system at these clinics. Surveys are administered by medical student volunteers at the conclusion of each patient encounter to capture information on the patient population and clinical services provided. This prospective data collection is intended to support clinical research and inform improvements in clinic operations.

The survey includes information regarding demographics, medical history, prescription history, chief complaints, prior diagnoses, and social history. The data analyzed in this study included all surveys completed between 2/19/17 and 7/9/23. The survey protocol is IRB approved and overseen by the research coordinator on the SCC executive council. Approximately 2,500 patients are seen across the preceptor clinics each year, and approximately one-third of these encounters are captured in the surveys.

The student-run clinics operated by this medical school provide a broad range of services including comprehensive primary and urgent care visits at preceptor-led clinic sites as well as targeted pop-up services such as tuberculosis screening, sexually transmitted disease screening, and vaccine administration at pop-up clinics. The survey described above is exclusively administered at preceptor clinic sites, which are located within shelters across the city. Specifically, the REDCap survey data included patients from four clinics: a men’s inpatient substance use recovery center, a women’s inpatient substance use recovery center, a co-ed shelter, and a men-only shelter.

Most of the preceptor clinics operate on a walk-in basis. However, the two clinics located in substance use recovery centers require scheduled appointments as they function as screening clinics for admission into the inpatient treatment programs. Because these clinics are located within shelters, the patients they serve are unhoused and rely on these facilities for accommodation. As a result, this clinical setting represents a distinct patient demographic in which all individuals are affected by the social determinants of health associated with housing instability, regardless of any additional factors such as a history of incarceration.

The surveys are typically completed by first- or second-year medical student volunteers following each patient encounter, based on information recorded during the visit. Only a limited number of fields are required by the software for survey completion including the fields of clinic site, date, and chief complaint. A visit may occur without a corresponding survey being submitted. Clinical care is primarily delivered by first-year medical students under the supervision of a fourth-year medical student and a resident or attending preceptor. The presence of a resident or attending allows for the provision of both over-the-counter and prescription medications to patients.

This study aimed to evaluate differences in chief complaints and medication distribution between patients with and without a reported history of incarceration. Only surveys in which respondents answered “yes” or “no” to the incarceration history question were included in the analysis. Surveys with missing responses in this field were excluded. Chief complaints were recorded as free-text entries and subsequently coded by the researchers. Complaints were grouped into broad categories corresponding to common body system complaints, such as musculoskeletal pain, psychological disorders, and respiratory complaints. Clinical judgement was used to assign each complaint to the appropriate category, resulting in over 19 distinct categories. Two researchers independently coded the free-text chief complaints to establish intercoder reliability. Discrepancies between coders were resolved by a third researcher. It was common for patients to be assigned multiple chief complaints.

Each complaint category was then compared with medications typically prescribed for that respective complaint, based on standard clinical practice. Medication data were collected from predefined checkbox options in the survey, as free-text responses were not permitted. The available medication categories included ACE inhibitors/calcium channel blockers/beta blockers; diuretics, antibiotics or antivirals, diflucan/antifungals, inhaled bronchodilator/inhaled steroid, insulin/oral antidiabetic drugs, psychiatric medications, NSAIDs (not acetaminophen), statin/lipid lowering drugs, antacid/PPI, allergy medication, cough/cold medication, topical steroid, smoking cessation (patches, gum, Chantix), acetaminophen, gabapentin.

Both chief complaint and medication administered were analyzed in relation to prior incarceration history. If there were fewer than *n* = 10 patients who received a medication for a specific chief complaint, or there was no medication grouping on the survey related to the chief complaint, the grouping was not included in the analysis.

Descriptive analyses were conducted using SPSS Statistics, with chi-squared tests used to evaluate associations and significance defined as *p* < 0.05. Cross tabulations were performed for each chief complaint by incarceration status. When the expected cell count was less than 5, a Fisher’s exact test was used to calculate the p-value. For any statistically significant association, logistic regression was performed to assess potential confounding of gender (male vs. female), race (person of color vs. non-person of color), and age (age < 65 or age > = 65). Medication prescription served as the dependent variable for the logistic regression models.

## Results

From 2/19/17 to 7/9/23, approximately 2,035 patients across New Orleans’ student-run clinics had a REDCap survey completed following their visit. Of these, 969 patients self-reported either a positive or negative history of prior incarceration while 1,066 either declined to answer or the information was not obtained. Of the 969 respondents, 692 (71%) reported a history of incarceration, and 277 (29%) reported no history of prior incarceration. Individuals who did not respond to the question about incarceration history were excluded from the analysis.

Among patients reporting a history of incarceration, 78% were male compared with 74% among those without a history of incarceration. The median age was 45 years old for formerly incarcerated patients and 48 years old for non-formerly incarcerated patients. Racial composition differed slightly between the groups with formerly incarcerated patients being 53% White and 38% Black, whereas non-incarcerated patients were 63% White and 32% Black.

**Fig. 1:**
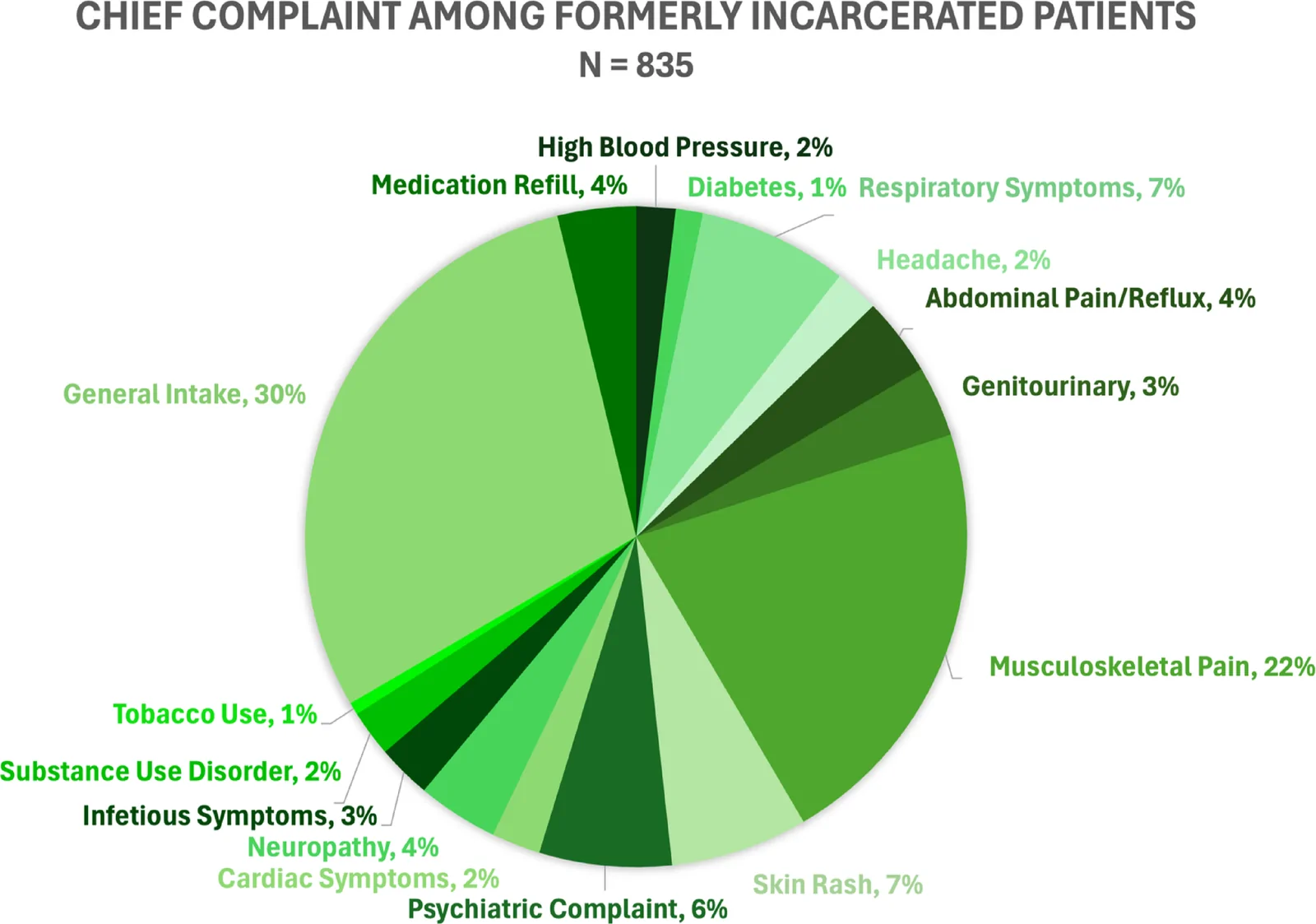
Distribution of Chief Complaints Among Formerly-Incarcerated Patients (*n* = 835). General intake (30%) and musculoskeletal pain (22%) represented the most common reasons for encounter

**Fig. 2:**
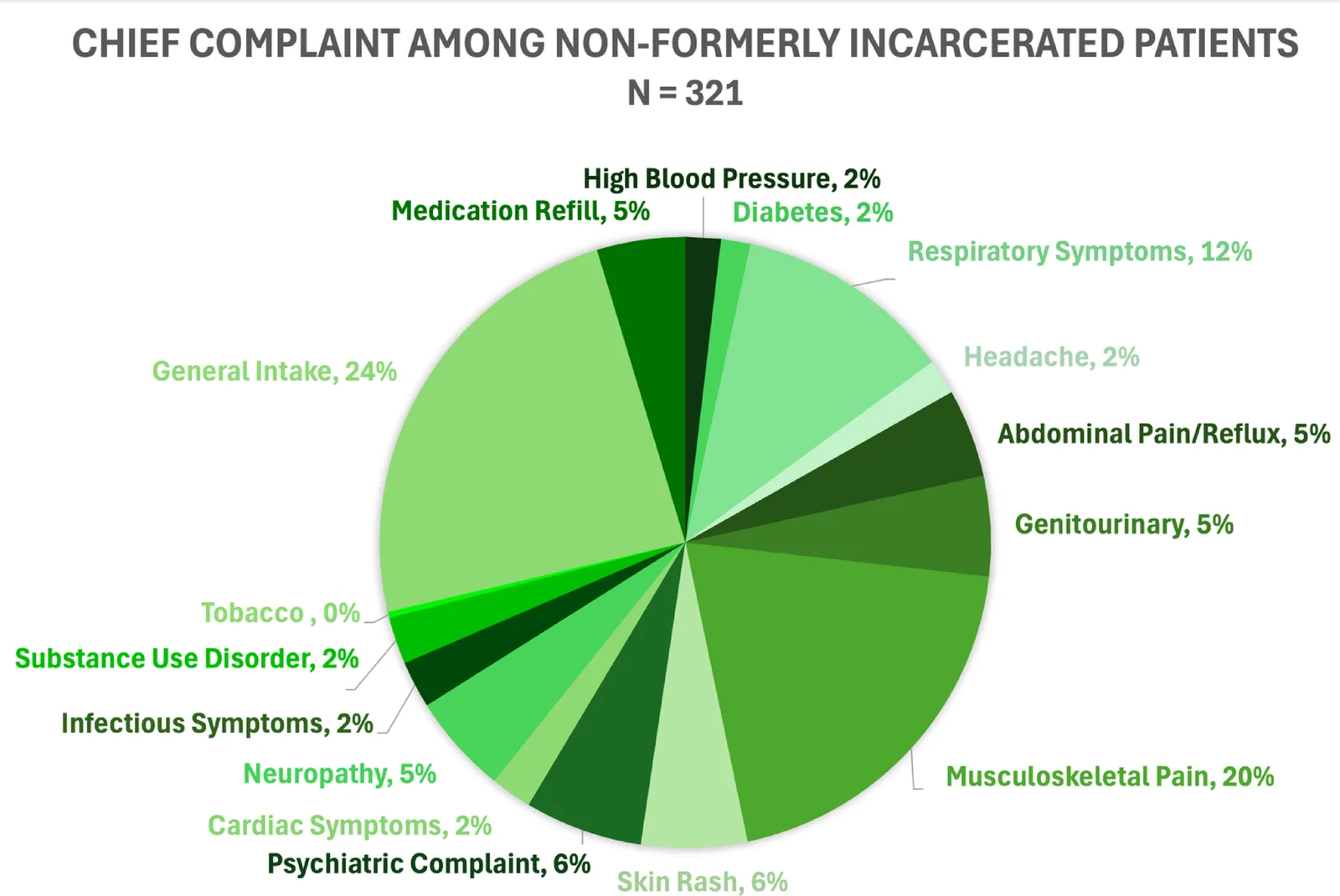
Distribution of Chief Complaints Among Non-Formerly Incarcerated Patients (*n* = 321). General intake visits (24%) and musculoskeletal pain (20%) represented the most common reasons for encounter

The analysis focused on three primary survey variables: chief complaint, medication prescribed, and incarceration history. Overall, there was no significant difference in chief complaints between formerly incarcerated versus non-formerly incarcerated patients presented (*p* = 0.199). Similarly, chi-square analysis of overall medication administration revealed no significant difference between groups (*p* = 0.920). Presenting concerns are summarized in Image 1. General intake evaluation, musculoskeletal pain, and upper respiratory symptoms were the most common complaints in both groups. Additionally, 17.6% of all patient surveys contained more than one chief complaint (Figs. 1 and 2).

When examining specific chief-complaint and medication pairings, no significant differences were observed for cardiovascular complaints treated with anti-hypertensives revealing *p* = 0.655 (OR = 0.600, 95% CI [0.090, 3.986]), musculoskeletal complaints treated with acetaminophen *p* = 0.527 (OR = 1.393, 95% CI [0.498, 3.898]) or NSAIDs *p* = 0.213 (OR = 1.531, 95% CI [0.781, 3.000]), high blood sugar complaints treated with diabetic medications either oral or insulin *p* = 1.000 (OR = 0.762, 95% CI [0.110, 5.283]), or reflux/epigastric pain treated with proton pump inhibitors *p* = 0.584 (OR = 0.714, 95% CI [0.214, 2.388]). Analysis of psychiatric complaints treated with psychiatric medications approached significance *p* = 0.080 (OR = 0.388, 95% CI [0.132, 1.141]), suggesting a potential trend worth further investigation.

A statistically significant difference was identified among patients presenting with respiratory symptoms such as self-reported “cough,” “runny nose,” or “cold symptoms,” with formerly incarcerated patients being less likely to receive cough and cold medication with *p* = 0.002 (OR = 0.201, 95% CI [0.068, 0.593]). This significance holds true even when adjusted for the 7 tests used with the Bonferroni correction, where significance is maintained as long as *p* < 0.0071. For the antihypertensive and diabetic medication groups, cells had expected count less than five, so a Fisher’s exact test was used to evaluate significance.

To assess for potential confounders, a logistic regression was performed for patients with respiratory symptoms using prescription of cough and cold medication as the dependent variable. After adjusting for gender (male versus female), race (person of color versus non-person of color), and age (age < 65 versus age > = 65), prior incarceration remained the only significant predictor of receiving these medications. Patients with a history of incarceration had substantially lower odds of being prescribed cough and cold medications. Gender, race, and age were not statistically significant, likely due to wide confidence intervals and limited sample size. Overall, the model was statistically significant and explained approximately 20% of the variability in medication prescribing (Table 1).

**Table 1:** Percentage of Formerly-Incarcerated (FI) and Non-Formerly-Incarcerated (NFI) Patients who Received Prescribed Medication for a Specific Chief Complaint (*n* = 969). The difference in FI vs. NFI patients with a chief complaint of upper respiratory symptoms who were prescribed cough and cold medications was statistically significant with *p* = 0.002

Chief Complaint	Medication Given	Total Chief Complaints			Prescribed Medication			% Prescribed Medications		*P*-value
		FI	NFI	Total	FI	NFI	Total	FI	NFI	
High Blood Pressure	Anti-Hypertensive	16	6	22	4	3	7	25	50	*p* = 0.655
MSK Pain	NSAID	180	64	244	54	14	68	30	21.8	*p* = 0.213
MSK Pain	Tylenol	180	64	244	19	5	24	10.6	7.8	*p* = 0.527
Diabetes	Diabetes Medication	11	5	16	4	3	7	36.4	60	*p* = 1.00
Psychiatric Complaint	Psychiatric Medication	54	20	74	24	9	33	44.4	45	*p* = 0.080
Reflux or Abdominal Pain	PPI	32	15	47	11	6	17	34.4	40	*p* = 0.584
**Upper Respiratory Symptoms**	Cough and Cold Medications*	**61**	**37**	**98**	**6**	**13**	**19**	**9.8**	**35.1**	*p* = 0.002

## Discussion

In this study of 2,035 patients who presented to student-run clinics in New Orleans, no significant difference was observed in presenting chief complaints between formerly incarcerated and non-formerly incarcerated patients. While previous research has documented a significantly higher prevalence of chronic diseases among incarcerated patients, such as hypertension, asthma, arthritis, cervical cancer, and hepatitis [5], chronic disease burden and health care utilization after release is less established. Recent evidence suggests that this chronic disease burden extends beyond incarceration, with an increased risk of chronic lung disease, mental health conditions, substance use, geriatric syndrome, and poor self-rated health in patients who had experienced any incarceration, even after socioeconomic adjustment [15]. Although our study survey design does not provide longitudinal data to directly assess chronic disease burden, the findings indicate no notable differences in chief complaints related to chronic conditions such as hypertension, asthma, or diabetes between formerly incarcerated and non-formerly incarcerated patients.

When interpreting the significance of these findings, it is important to consider that the comparison group is not representative of the general population. Most patients attending these student-run clinics are experiencing homelessness or substance misuse, which are themselves significant social determinants of health associated with a high burden of chronic disease [16, 17, 18]. This context may explain the lack of significant difference between formerly incarcerated and non-formerly incarcerated patients’ chief complaints in these clinics. The findings do not suggest that prior incarceration has no impact on chronic disease, but rather, the comparison group already faces substantial health risks due to their existing socioeconomic disadvantage and barriers to healthcare.

Another key aspect of this study was the evaluation of medication prescribing patterns between formerly incarcerated and non-formerly incarcerated patients. Overall, no significant differences were observed in prescription rates between groups. Existing literature on medication distribution for recently released or formerly incarcerated patients is limited. One recent study of incarcerated populations reported substantially lower use of medications for chronic conditions in jails and state prisons compared with the non-incarcerated population [10]. However, these findings are not directly comparable to this study, which examines a post-incarceration population. This gap in the literature was a primary motivation for the study, and the authors hope that these findings contribute to a better understanding of the healthcare experiences of formerly incarcerated individuals, particularly regarding potential biases they may face after release.

In addition to the findings mentioned above, formerly incarcerated patients presenting with respiratory symptoms were significantly less likely to receive cough and cold medication. These medications included over-the-counter options such as cough syrups and combination formulations containing dextromethorphan like DayQuil/NyQuil. Literature addressing potential biases in medication prescription for incarcerated or formerly incarcerated individuals is limited. However, a 2014 study found that 42% of recently released male inmates experienced discrimination by healthcare workers based on their criminal record [19]. This finding suggests that bias may contribute to the disparities in medication prescribing rates seen in these clinics. Supporting this interpretation, logistic regression analyses ruled out gender, age, and race as significant cofounders.

One potential contributor to the observed prescribing disparity may be the perception that formerly incarcerated patients have higher rates of substance use disorders compared to the general population. For example, a 2019 report from the Prison Policy Initiative found that approximately 8% of people aged 12 and older in the United States met criteria for a substance use disorder compared with 41% of those arrested and 32%-49% of those in federal or state prisons [20]. Such statistics, combined with prevailing attitudes in clinical medicine, may influence prescribing practices in these clinics particularly for cough and cold medications which often contain dextromethorphan (DXM), a substance known to be subject to misuse [21]. While provider bias is a plausible explanation for this finding, an alternative explanation is that the survey does not capture whether a medication was offered but declined by the patient, representing a limitation of the current study design.

Overall, this study contributes to the growing field of research on the health disparities faced by formerly incarcerated individuals as they navigate the healthcare system after their release. These findings emphasize the importance of educating student-run clinic physicians and medical student volunteers about both the chronic disease burden and potential biases faced by this population. Additionally, the study highlights a notable gap in the literature regarding medication prescribing practices for formerly incarcerated patients, emphasizing the need for more research in this area.

## Limitations

Several limitations of this study must be acknowledged. The primary limitation relates to the survey used to collect patient data. The survey captures only chief complaints and does not include questions to obtain the final diagnosis of the visit. As a result, analyses were based on the patient’s self-reported concerns rather than confirmed clinical diagnoses. This impacts our analysis, as there may have been instances in which the chief complaint and final diagnosis differed in a way that would affect medication distribution. This, in turn, may cause our analysis to present an incomplete picture. Alternative data fields such as “past medical history” and “new diagnosis” were considered to enhance the analysis, however these were incomplete for a majority of patients. Future survey improvements, including a required final diagnosis field, would allow for more accurate data capture and interpretation. Additionally, the implementation of an electronic medical record system in these clinics could further reduce data limitations but may be cost prohibitive.

Additional limitations arise from the process of survey data collection. Surveys were primarily completed by first-year medical students with varying levels of clinical experience who assisted patients in answering health-related questions. This may have contributed to incomplete responses, particularly regarding incarceration history and resulted in a reduced analyzable sample size. Furthermore, patients presenting with focused complaints may have received limited histories, which could have prevented full survey completion. Finally, these surveys are specific to student-run clinics organized by one medical school in New Orleans, which may limit the generalizability of the findings. Valuable information could be obtained if this analysis were to be replicated at other student-run clinics.

## Conclusion

In conclusion, no differences were observed in the presenting chief complaints or overall medication prescription rates between formerly incarcerated and non-formerly incarcerated patients in our student-run clinics. However, formerly incarcerated patients presenting with respiratory symptoms were less likely to receive cold and cough medications, suggesting a potential bias in prescribing practices. Recognizing and addressing such biases is essential to improving the quality of care provided to this population. Further research is needed to evaluate medication prescribing patterns among formerly incarcerated patients in other settings. These findings may also inform educational initiatives for physician and student volunteers, fostering awareness of potential bias and promoting more equitable care.
